# Association between depressive symptoms of mothers and eating behaviors of school-going children in Urban Bangladesh: A cross-sectional study

**DOI:** 10.1186/s12905-023-02584-w

**Published:** 2023-08-18

**Authors:** Sharmin Sultana, Faisal Muhammad, ABM Alauddin Chowdhury, Tasmia Tasnim, Md. Imdadul Haque, Abul Hasan BakiBillah, Md. Kamrul Hossain, Sanjana Zaman, Mohammad Delwer Hossain Hawlader, Moniruddin Chowdhury

**Affiliations:** 1 Department of Public Health, Daffodil International University, Daffodil Smart City, Birulia, Savar, Dhaka, 1216 Bangladesh; 2 Department of Public & Community Health, Faculty of Medicine & Health Sciences, Frontier University Garowe, Puntland, Somalia; 3 Otu Institute of Research and Training, Kano, Nigeria; 4 Department of Nutrition and Food Engineering, Daffodil International University, Daffodil Smart City, Birulia, Savar, Dhaka, 1216 Bangladesh; 5 Department of Health Economics, Faculty of Allied Health Sciences, Bangladesh University of Health Sciences (BUHS), Dhaka, 1216 Bangladesh; 6 Department of General Educational Development, Daffodil International University, Daffodil Smart City, Birulia, Savar, Dhaka, 1216 Bangladesh; 7 Department of Public Health, North South University, Dhaka, 1229 Bangladesh; 8 Faculty of Medicine, AIMST University, Bedong, Kedah, 08100 Malaysia

**Keywords:** Depressive symptoms, Mother, Eating behaviors, School-going children, Dhaka, Bangladesh

## Abstract

**Objective:**

This study aimed to investigate the association between depressive symptoms among mothers and the eating behaviors of their school-going children in Urban Bangladesh.

**Materials and methods:**

This analytical cross-sectional study was conducted in the context of the urban area of Bangladesh. A multistage sampling technique was applied to select 324 children’s mothers in Dhaka City. Data were collected from both city corporation settings in Dhaka, Bangladesh. Semi-structured questionnaires were used in this study. We estimated the depressive symptoms among mothers using the Zung Self-Rating Depression Scale. We examined the association of mothers of school-going children’s socio-demographic variables and eating behaviors of school-going children with their mother’s depression by using chi-square and evaluating the impact of these variables on mothers’ depression through univariate and multivariate binary logistic regression.

**Results:**

In our study, 57.7% of the mothers of school-going children had depressive symptoms, and 42.3% had no depressive symptoms. The study explored that consuming fewer vegetables (AOR = 0.237, 95% CI: 0.099–0.569), taking fewer fruits (AOR = 0.177, 95% CI: 0.093–0.337), and interestingly, taking fast food less than 4 days per week (AOR = 3.024, 95% CI: 1.517–6.031) were significantly associated with mothers’ depressive symptoms.

**Conclusion:**

Mothers with depressive symptoms of school-going children in Dhaka city are alarmingly high as a grave concern. The eating behaviors of children are associated with their mothers’ depressive symptoms. With an aim to build rigorous awareness on depression and child’s healthy eating behaviors, it is imperative to arrange health education and awareness related programs.

## Introduction

Mental Health is labeled a neglected public health issue though more than 450 million people worldwide suffer from various mental illnesses [1]. Globally, depression is identified as a common mental illness and one of the leading causes of disability [2]. As per the estimation, about 264 million people are affected by depression worldwide [3]. Depression, in terms of morbidity and disability, is more deadly in developing countries [4]. Developing countries, for example, Bangladesh, reported that mental disorders (stress, anxiety, and depression) are higher [5], and the prevalence of mental disorders varied from 6.5 to 31.0% among adults [6]. The overall prevalence of mild and moderate depression is 17.9%, and 5.4%, respectively, among Bangladeshi people [7]. The data reflects that about 7 million people in Bangladesh experience depressive and anxiety disorders.

Several studies showed that mothers of young children are at considerable threat for psychological well-being-related troubles, the estimated national prevalence of depressive symptoms was ranging from 10.0 to 50.0% [8–11]. Moreover, Depression is significantly connected with less positive parenting in mothers. The findings are mostly associated with mothers rather than fathers [12]. With grave concern, a study reported a relationship between unhealthy eating behavior and consumption of a low-quality diet and depression or poor mental health [13].

Additionally, this insufficient diet, poor in both quality and quantity has been one of the proven causes of extreme levels of malnutrition among children [14]. Maternal depression has lately emerged as a risk factor for poor health outcomes in children [15]. Importantly, maternal mental health plays a major contributing role to the nutritional status of school-going children; just because they are often found restless and reluctant to the required level of nutritional intake [9, 10, 16–18]. Some of the existing studies have indicated a relationship between maternal depression and children’s diet [19, 20]. Depressed mothers may lack the motivation and energy to seek out and prepare healthy meals. Depression has been linked to a decline in maternal-child bonding: depressed women’s moods may interfere with their understanding of their child’s nutritional needs. Multiple or recurring episodes of maternal depression symptoms in preschool or older children are likely to jeopardize mother-child feeding and other behavior patterns [15]. Moreover, some studies have revealed associations between mothers’ depression symptoms and their children’s lower consumption of essential foods for example vegetables, and fruits [21, 22].

Epidemiological studies and data related to the prevalence of maternal depression and its impact on children’s health and nutritional outcomes are quite limited in Bangladesh. A recent study among the mothers of school-going children in Dhaka identified that the depressive symptoms of mothers are associated with some factors like sickness, injury, and unfriendly relationships between mothers and children [23]. In addition, in Bangladesh, an urban slum community focused a study on maternal mental health and child nutritional status [24].

To our best level of knowledge, no study has yet concentrated on the association between mothers’ depressive symptoms and the eating behaviors of their school-going children in Bangladesh. Therefore, the study aimed to investigate whether the depressive symptoms among mothers are associated with the eating behaviors of their school-going children in Urban Bangladesh.

## Materials and methods

### Design

We conducted this analytical cross-sectional study in Dhaka City, Bangladesh from June to December 2019. Dhaka is the capital city comprising two city corporations; the North City Corporation and the South City Corporation. For collecting data, we randomly selected 6 public and 6 private schools from both city corporations of Dhaka City.

### Study participants and sampling

The sample size was calculated using the following formula:

$$\text{n}=\frac{{\text{z}}^{2}\text{p}(1-\text{p})}{{\text{d}}^{2}}$$

Where, n = desired sample size; z = 1.96 at 95% CI; p = prevalence of antepartum depressive symptoms in Bangladesh = 18% [25]; and d = sampling error (4.18%). Thus, the calculated sample size was 324 (n).

We randomly selected mothers of school-going children studying both in public and private schools of Dhaka city from 12 different schools. The encompassing inclusion criteria were the mothers of children (class V- VIII) who studying in the same school for at least 6 months. Exclusion criteria were mothers not willing to partake in the study. However, we chose the juror students studying in class V-VIII as the country’s Ministry of Education is responsible for implementing policy for primary education and state-funded schools at a local level up to class VIII that has been recently considered as compulsory and included a standardized public exam held in class VIII called Junior School Certificate examination. There is no choice of specialized subjects offered to students under this curriculum up to class VIII, and more especially students up to this stage pursue their education fully under the parents’ control [26].

**Fig. 1:**
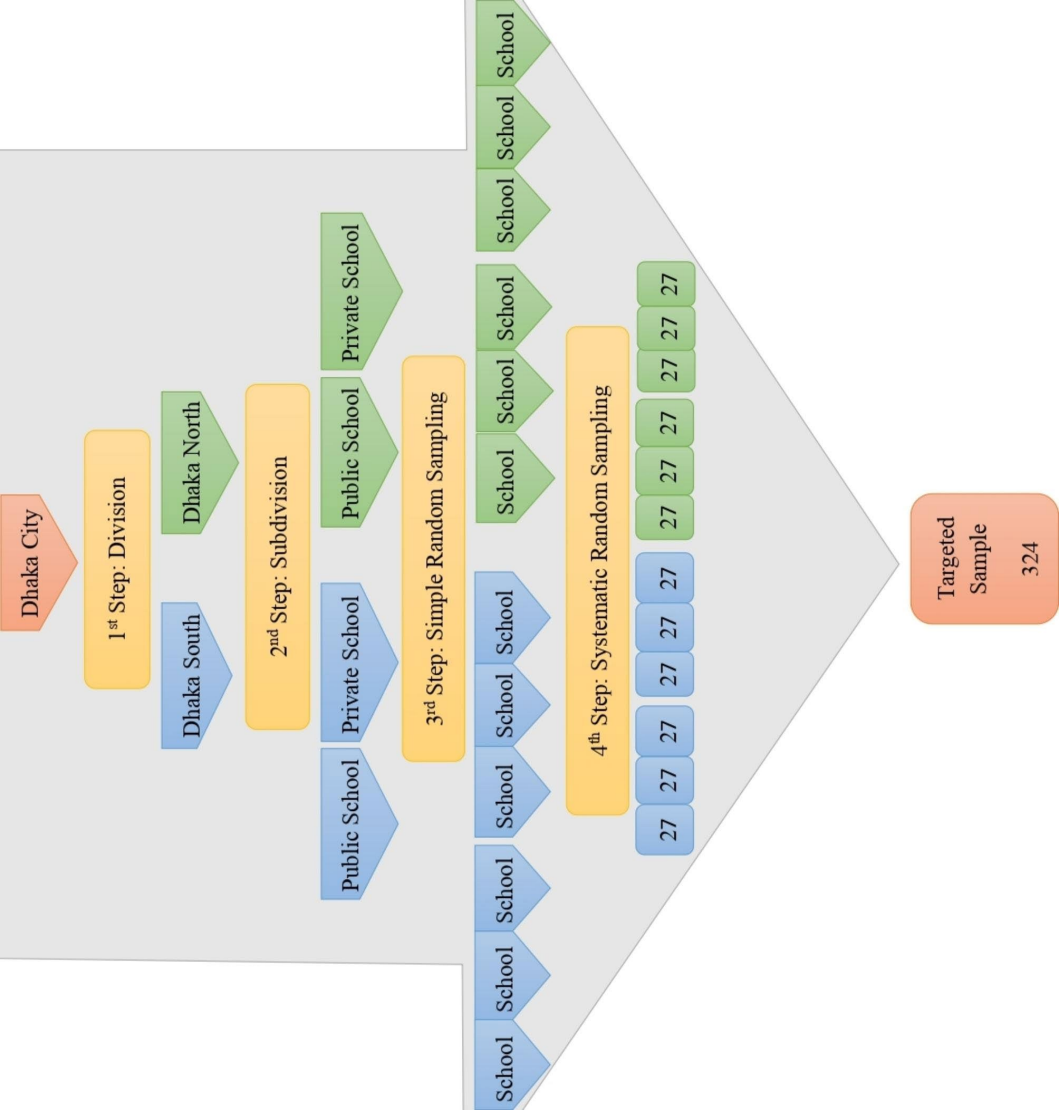
Multistage sampling technique

The study applied a multi-stage randomized sampling technique (figure-1). In the first step, we selected the administratively divided two city corporations from Dhaka city: North and South, and in the second step, we randomly selected six public schools and six private schools from each city corporation to minimize any potential bias in terms of the socio-economic status of the participants. Based on that categorization, we collected a list of public and private schools. Subsequently, the study collected a list of class V-VIII students from each school from which the systematic sampling procedure drew the targeted samples. Notably, around one-eighth of the participants in the sampling frame were unavailable during the data collection period, so the next student who fulfilled the inclusion criteria was selected. The absence of such a portion of the participants sounds certainly odd. Here, it needs to be noted that there was no funding for the study, and we found school grounds convenient, especially during the tiffin period to collect information. However, for more than three-quarters of the cases, mothers were not present in the school with their children who were genuinely our participants by inclusion criteria. Just because, students in those cases reached at the either by themselves or by their servants, caregivers, or drivers.

### Data collection procedures

With the assistance of the mentor, the Principal Investigator (PI) developed the questionnaires considering the socio-demographic characteristics, and eating behaviors of children. The mothers were interviewed to obtain the children’s eating behavior data. Five relevant experts from the wide range in the professionals and academia like psychologists, nutritionists, epidemiologists, and public health researchers reviewed the questionnaires and incorporated their inputs. The questionnaires were firstly prepared in English, then translated to Bengali, again back-translated to English, and checked by the same reviewers to minimize potential errors and maintain the context and sequences of the questions. The study then pretested the Bengali version of the questionnaire in six non-sampling study areas (for the issue with reporting bias), getting feedback on the questions’ acceptability, appropriateness, and sequencing. Accordingly, after making the necessary modifications and corrections experienced from the pretest, the questionnaire was finalized. Since only one interviewer (the PI) inter-rater reliability check was not deemed necessary. Face-to-face interviews were conducted to collect the data by using semi-structured questionnaire. In terms of the completeness and accuracy of the data, we checked thoroughly and maintained a logbook/notebook to make any necessary changes daily during the data collection.

### Outcome variables

The rate of depressive symptoms was estimated by the validated Zung Self-Rating Depression Scale (SDS), which quantified the depressed state of a patient, including 20 items, and scored on a scale of 1–4. The SDS is convenient for identifying clinically significant depressive symptoms in adults [27, 28]. It was validated, shown to be efficient, and thereafter used extensively throughout the world for screening the psychometric characteristics of general adult and senior individuals in a non-clinical and community environment [29–31]. The weighted scores were considered up to 100%, with 25–49 categorized as no depression, 50–59 as mild depression, 60–69 as moderate depression, and 70 or above as severe depression. The reliability score or Cronbach’s alpha for the questionnaires (20 items) was 0.894. Notably, our dependent variable was depressive symptoms found from the SDS scale and the independent variables were socio-demographic characteristics and eating behaviors as indicated in Table 1.

### Statistical analysis

Data were coded, entered, and cleaned by Statistical Package for the Social Sciences (SPSS) Version 22. We used descriptive statistics e.g. frequencies and proportions to summarize the data. The Cronbach’s alpha coefficient was calculated to check the reliability of the English version of the SDS.

We performed the Chi-square test to measure the degrees of association between the outcome and the independent variables. Notably, the dependent variable is depressive symptoms and the independent variables are socio-demographic characteristics and eating behaviors. Additionally, we conducted multivariate analyses using binary logistic regression to observe the association between the mother’s depressive symptoms and other socio-demographic and eating behaviors factors. We set the statistical significance (p-value) of < 0.05. To assess the strength of the association, we used adjusted odds ratios (AOR) and their 95% confidence intervals (CIs) as indicators.

## Results

### Socio-demographic characteristics of the participants

More than half of the mothers of school-going children (54.30%) were ≥ 40 years old, with mean age (± SD) of 39.5 ± 4.9 years. Most participants (85.50%) were Muslim, and more than two-thirds (67.00%) of the mothers were housewives. More than half of them (53.10%) had girl children (Table 1).

It was observed that apart from age, all other socio-demographic factors, such as religion (χ2 = 6.322); occupation (χ2 = 59.446); and gender of children (χ2 = 3.869) had a significant association with mothers depression at 5% level of significance as p value less than 0.05 (Table 2).

**Table 1:** Socio-demographic characteristics of the respondents (n = 324)

Characteristics		Frequency	Percent
Age **(Mean ± SD: 39.5 ± 4.9)**	≥ 40	176	54.3
	< 40	148	45.7
Religion	Muslim	277	85.5
	Non-Muslim	47	14.5
Occupation	Employed	107	33.0
	Housewife	217	67.0
Gender of child	Boy	152	46.9
	Girl	172	53.1
Eat vegetables in a week	≥ 4 days	89	27.5
	< 4 days	235	72.5
Eat fruits in a week	≥ 4 days	125	38.6
	< 4 days	199	61.4
Eat fast foods in a week	≥ 4 days	206	63.6
	< 4 days	118	36.4
Eat sweets in a week	≥ 4 days	278	85.8
	< 4 days	46	14.2
Consume soft drinks in a week	≥ 4 days	205	63.3
	< 4 days	119	36.7

SD = standard deviation

### Eating behaviors of children and depressive symptoms among mothers

The study identified that more than half (57.70%) of mothers had depression. Child’s dietary habits except having sweets, taking vegetables (χ2 = 52.040), fruits (χ2 = 54.158), fast foods (χ2 = 42.499), and soft drinks (χ2 = 22.466) had the significant association with the mother’s depression at 5% level of significance as p value less than 0.05 (Table 2).

**Table 2:** Association between the dependent variable and independent variables (n = 324)

Characteristics		Depression Status [No (n = 137; 42.3%); Yes (n = 187; 57.7%)]		χ2 value
		No n (%)	Yes n (%)	
Age (Years)	≥ 40	76 (55.5)	100 (53.5)	0.127
	< 40	61 (44.5)	87 (46.5)	
Religion	Muslim	125 (91.2)	152 (81.3)	6.322**
	Non-Muslim	12 (8.8)	35 (18.7)	
Occupation	Employed	13 (9.5)	94 (50.3)	59.446**
	Housewife	124 (90.5)	93 (49.7)	
Gender of child	Boy	73 (53.3)	79 (42.2)	3.869**
	Girl	64 (46.7)	108 (57.8)	
Eat vegetables in a week	≥ 4 days	9 (6.6)	80 (42.8)	52.040**
	< 4 days	128 (93.4)	107 (57.2)	
Eat fruits in a week	≥ 4 days	21 (15.3)	104 (55.6)	54.158**
	< 4 days	116 (84.7)	83 (44.4)	
Eat fast foods in a week	≥ 4 days	115 (83.9)	91 (48.7)	42.499**
	< 4 days	22 (16.1)	96 (51.3)	
Eat sweets in a week	≥ 4 days	120 (87.6)	158 (84.5)	0.623*
	< 4 days	17 (12.4)	29 (15.5)	
Consume soft drinks in a week	≥ 4 days	107 (78.1)	98 (52.4)	22.466**
	< 4 days	30 (21.9)	89 (47.6)	

^*^P-value < 0.5

^**^P-value < 0.05

### Predictor’s mothers’ depression

Religion was significantly associated with the status of depression among mothers in the unadjusted model [OR (95% CI) = 0.417 (0.208–0.837)]; it became insignificant [OR (95% CI) = 0.752 (0.286–1.981)] after adjusted by others independent variables. Participants’ occupation was strongly significant in both unadjusted [OR (95% CI) = 9.641 (5.087–18.270)] and adjusted model [OR (95% CI) = 6.506 (2.948–14.357)]. Also, the study revealed that there was no significant difference between having a male child and mother’s depression; [OR (95% CI) = 0.641 (0.412–0.999)]. Even, after adjustment, the association remains the same [OR (95% CI) = 0.705 (0.392–1.268)] (Table 3).

A child’s eating vegetables < 4 days in a week was associated with mother’s depression and the relationship was strongly significant in both unadjusted [OR (95% CI) = 0.094 (0.045–0.196)] and adjusted model [OR (95% CI) = 0.237 (0.099–0.569)]. Similarly, fruits intake < 4 days in a week was strongly significant in both unadjusted [OR (95% CI) = 0.144 (0.084–0.250)] and adjusted analysis [OR (95% CI) = 0.177 (0.093–0.337)]. Intriguingly, we discovered that in both the unadjusted and adjusted models, the mother whose child eats fast food less than 4 days per week had a more than three times higher likelihood of being depressed [OR (95% CI) = 5.514 (3.218–9.451)] and the adjusted model, the risk was found a little lower [OR (95% CI) = 3.024 (1.517–6.031)]. In addition, consuming child’s soft drinks was significantly associated with the mothers’ depression in the unadjusted model [OR (95% CI) = 3.239 (1.972–5.321)] but became insignificant [OR (95% CI) = 1.267 (0.654–2.452)] after adjusted with others independent variables (Table 3).

**Table 3:** Unadjusted and adjusted analysis (binary logistic regression) of dependent (depression yes or no) and independent variables (n = 324)

Variables		Unadjusted model			Adjusted model	
		OR		CI	OR	CI
Religion	Muslim	0.417		0.208–0.837**	0.752	0.286–1.981
	Non-Muslim	Reference			Reference	
Occupation	Employed	9.641		5.087–18.270**	6.506	2.948–14.357**
	House wife	Reference			Reference	
Gender of child	Boy	0.641		0.412–0.999*	0.705	0.392–1.268*
	Girl	Reference			Reference	
Eat vegetables in a week	< 4 days	0.094	0.045–0.196**		0.237	0.099–0.569**
	≥ 4 days	Reference			Reference	
Eat fruits in a week	< 4 days	0.144	0.084–0.250**		0.177	0.093–0.337**
	≥ 4 days	Reference			Reference	
Eat fast foods in a week	< 4 days	5.514	3.218–9.451**		3.024	1.517–6.031**
	≥ 4 days	Reference			Reference	
Consume soft drinks in a week	< 4 days	3.239	1.972–5.321**		1.267	0.654–2.452*
	≥ 4 days	Reference			Reference	

OR = Odds Ratio; CI = Confidence Interval

^*^P-value < 0.5

^**^P-value < 0.05

## Discussion

The purpose of this study was to investigate the association between depressive symptoms of mothers and eating behaviors of school-going children in Urban Bangladesh.

In our study, 57.7% of the mothers of school-going children had depressive symptoms. This finding is consistent with a previous investigation conducted in Dhaka, Bangladesh, which found that 39.4% of postpartum mothers had depression [32]. Additionally, an Indian study found that 38.3% of mothers of children with neurodevelopmental problems reported having depressive symptoms [33].

Mothers must take into account the food supply, which is connected to agricultural-food systems that influence the demand for and usage of food, while making decisions about what to feed their children [34]. This study discovered a link between children’s eating habits and their mother’s depression symptoms. In line with this finding, a study of UK mothers found a link between the mother’s depressive symptoms and their child’s eating habits [35]. Notably, our study identified a high correlation between the mother’s depression and the children’s lower intake of fruits and vegetables. This finding aligns with a recent study that examined the relationship between maternal depressive symptoms and children’s limited intake of fruits and vegetables [21]. Another longitudinal study illustrated the association between mothers’ depressive symptoms and their kids’ reduced intake of nutritious foods such as vegetables, fruits, milk, and juice, etc. [22]. This implies that maternal depression may possibly impact food purchase patterns and their preparation, leading to a decreased availability and consumption of nutritious foods in the household. Additionally, it suggests that interventions targeting maternal mental health may also have a positive impact on improving children’s dietary habits.

The current study also revealed that the occupational status of mothers are connected with frequency of depressive symptoms. In a previous study, caregivers with high-level depressive symptoms had a significantly higher prevalence of unemployment than caregivers with low-level depressive symptoms [36]. Another recent study also demonstrated that maternal depression was strongly associated with a range of adverse economic outcomes, including not being employed, material hardship, and poverty [37]. Previously, researchers have asserted that poverty or having low financial resources is specifically related to maternal depression and may further contribute to the limited access to nutritious foods for both the mother and child. This highlights the complex interplay between socioeconomic factors, mental health, and dietary habits, emphasizing the need for comprehensive interventions that address these interconnected issues to promote optimal nutrition and well-being in children [38].

However, this study found no association between mother’s depressive symptoms and children consumption of soft drinks. Interestingly, our study also discovered that mothers with depressive symptoms had children who consumed less fast food. In contrast, a post-hoc study of the large birth cohort survey revealed that children with depressive mothers consumed more sweetened beverages and fewer fruits and vegetables, although fast food intake was unaffected [39]. This suggests that there may be other factors at play when it comes to children’s dietary choices, and that maternal depressive symptoms may not be the sole determinant. Further research is needed to explore the complex relationship between maternal mental health and children’s dietary habits.

The level of mothers’ nutritional understanding of their children’s eating habits is the cause of the differences between the current findings and other findings. Some mothers bribe their kids with delicious delights like sugary snacks to get them to behave better [40]. Some mothers with poor nutritional knowledge may even actively promote their children’s obesity by feeding them unhealthy foods [41].

### Limitations of this study

The results of the current study should be interpreted with caution due to its cross-sectional design, which limits the ability to establish causality. Other factors, such as socioeconomic status and family dynamics, may also influence both maternal depression and children’s eating habits. Future research should consider longitudinal designs and explore the underlying mechanisms that contribute to the association between maternal depression and children’s eating behaviors. This will help develop targeted interventions to improve both maternal mental health and children’s dietary choices. The use of self reported measures is also a disadvantage because the responses may be subject to biases and inaccuracies. One other limitations of our study is that around one-eighth of the total students were observed as missing in the class during data collection. The next study participant in the sampling frame opted out in those cases. This could generate selection bias. It may be considered as a weakness of this study. In addition, due to the cross-sectional sampling design, this study determined adjusted associations rather than causality. Moreover, there was also a limitation in the measure of consistency because of the absence of inter-rater reliability.

### Conclusions and recommendations

We conclude that more than half of the mothers had depressive symptoms, which drew the public health concentration as a grave concern also, this study figured out that school-going children’s low consumption of vegetables, fruits, and fast food was substantially related to their mother’s depressive symptoms. With an aim to build rigorous awareness on depression and child’s healthy eating behaviors, it is imperative to arrange health education and awareness related programs. Practical interventions and social safety nets, especially for working mothers with minimal childcare support systems that help mitigate depression, must be integrated directly into maternal and child nutrition programs. Future study may be performed to understand better food-related decision-making and feeding methods concerning mothers’ mental health, their link with children’s growth and cognitive development, and to find modifiable risk factors for effective prevention and mitigation. Hence, a lack of adequate understanding regarding diet might be a possible risk for depression, although this was not investigated in the current study. The link between mothers’ depression and children’s fast food consumption remains unclear. More detailed and in-depth surveys may be required to investigate the background and motivations for the observed behavior of such cohorts.

## Data Availability

The datasets generated or analyzed during the current study are private because the authors have some reservations about making data publicly available but are available from the corresponding author upon reasonable request.

## Abbreviations

AOR: Adjusted Odds Ratios
CI: Confidence Interval
OR: Odds Ratio
PI: Principal Investigator
SD: Standard Deviation
SDS: Zung Self-Rating Depression Scale
UK: United Kingdom
